# The effects of conduction delay on temporal ordering in leaky integrate and fire neuronal networks

**DOI:** 10.1186/1471-2202-12-S1-P382

**Published:** 2011-07-18

**Authors:** Tony L Smith, Michal Zochowski

**Affiliations:** 1 Department of Applied Physics, University of Michigan, Ann Arbor, MI 48105, USA; 2 Department of Applied Physics, University of Michigan, Ann Arbor, MI, 48105, USA

## 

Neurons in the mammalian cortex have conduction latencies that can scale up an order of magnitude or more with myelination and axon radius. These latencies can be categorized into two groups: distance dependent latencies associated directly with axonic/dendritic length and distance independent latencies due to intrinsic delays on synapses themselves. This paper focuses on the distance dependence of conduction latency, attempting to elucidate its effects on the temporal ordering of the network activity patterns. We use various statistical measures to characterize the temporal patterning behavior of the networks: mean phase coherence, a pairwise measure of phase locking between neuron spike trains averaged over the network and synchronous bursting, which quantifies the coincidence of spikes as it deviates from a Poisson process.

We find that distance dependent delays and distance independent delays can lead to significantly different spatio-temporal patterning of network wide activity. The introduction of distance dependent delays into the network can drive temporal dynamics toward a more synchronous state. This synchronization enhancement can decrease as the topology of the network shifts towards a random graph and as the conduction speed moves out of an optimal range.

